# Cardiac-directed expression of a catalytically inactive adenylyl cyclase 6 protects the heart from sustained β-adrenergic stimulation

**DOI:** 10.1371/journal.pone.0181282

**Published:** 2017-08-02

**Authors:** Mei Hua Gao, N. Chin Lai, Dimosthenis Giamouridis, Young Chul Kim, Tracy Guo, H. Kirk Hammond

**Affiliations:** 1 VA San Diego Healthcare System, Department of Medicine, Division of Cardiology, San Diego CA, United States of America; 2 University of California, San Diego, Department of Medicine, Division of Cardiology, La Jolla CA, United States of America; University of Cincinnati College of Medicine, UNITED STATES

## Abstract

**Objectives:**

Increased expression of adenylyl cyclase type 6 (AC6) has beneficial effects on the heart through cyclic adenosine monophosphate (cAMP)-dependent and cAMP-independent pathways. We previously generated a catalytically inactive mutant of AC6 (AC6mut) that has an attenuated response to β-adrenergic receptor stimulation, and, consequently, exhibits reduced myocardial cAMP generation. In the current study we test the hypothesis that cardiac-directed expression of AC6mut would protect the heart from sustained β-adrenergic receptor stimulation, a condition frequently encountered in patients with heart failure.

**Methods and results:**

AC6mut mice and transgene negative siblings received osmotic mini-pumps to provide continuous isoproterenol infusion for seven days. Isoproterenol infusion caused deleterious effects that were attenuated by cardiac-directed AC6mut expression. Both groups showed reduced left ventricular (LV) ejection fraction, but the reduction was less in AC6mut mice (p = 0.047). In addition, AC6mut mice showed superior left ventricular function, manifested by higher values for LV peak +dP/dt (p = 0.03), LV peak -dP/dt (p = 0.008), end-systolic pressure-volume relationship (p = 0.003) and cardiac output (p<0.03). LV samples of AC6mut mice had more sarco/endoplasmic reticulum Ca2+-ATPase (SERCA2a) protein (p<0.01), which likely contributed to better LV function. AC6mut mice had lower rates of cardiac myocyte apoptosis (p = 0.016), reduced caspase 3/7 activity (p = 0.012) and increased *B-cell lymphoma 2* (*Bcl2)* expression (p = 0.0001).

**Conclusion:**

Mice with cardiac-directed AC6mut expression weathered the deleterious effects of continuous isoproterenol infusion better than control mice, indicating cardiac protection.

## Introduction

Substitution of Ala for Asp at position 426 in the catalytic core of adenylate cyclase type 6 (AC6), renders the molecule catalytically inactive, which impairs cyclic adenosine monophosphate (cAMP) generation, enabling separation of cAMP-dependent and cAMP-independent effects of AC6 in studies conducted in cultured cardiac myocytes [1],[2],[3],[4]. Transgenic mice with cardiac-directed AC6mut expression possess normal LV contractile response to isoproterenol, despite impairment of LV **β**-adrenergic receptor-stimulated cAMP production [5]. This was shown to be through the beneficial effects of AC6mut on Ca2+ handling, which are predominantly cAMP-independent [5]. The next logical question, which we address in the current study, is whether cardiac-directed expression of AC6mut might protect the heart from the deleterious consequences of sustained **β**-adrenergic receptor stimulation, which is often seen in association with heart failure.

The current study has a potential important clinical application. A randomized clinical gene transfer trial using intracoronary delivery of a modified adenovirus-5 vector encoding human adenylyl cyclase type 6 showed beneficial effects in patients with symptomatic heart failure with reduced ejection fraction was reported in 2016 [6]. In this trial, unaltered AC6, which generates cAMP, was used. There were no adverse effects attributable to increased cAMP generation seen in the trial. Even so, attenuating βAR responsiveness when treating heart failure may have advantages, and further refinement of the transgene seems prudent.

The goal of the current study was to perform mechanistic and translational studies of the effects of cardiac-directed expression of AC6mut specifically to determine how such animals would fare in the setting of sustained increases in **β**-adrenergic receptor stimulation, a condition frequently encountered in subjects with heart failure. Our hypothesis was that cardiac-directed expression of AC6mut would protect the heart from such deleterious effects, by governing cardiac responsiveness.

## Materials and methods

### AC6mut transgenic mice

AC6mut mice in congenic C57BL/6N background were used [5] and transgene negative siblings were used as controls. One hundred seven mice (44% female; 4.1±0.2 months-old; 24.3±0.4 grams) were used. Mouse genotyping was performed by PCR. We previously reported details regarding the generation of transgenic mice with cardiac-directed AC6mut expression [5]. The use of animals was in accordance with Association for Assessment and Accreditation of Laboratory Animal Care guidelines and was approved by the Institutional Animal Care and Use Committee of VA San Diego Healthcare System.

### Isoproterenol infusion

Osmotic minipumps (Alzet Model; DuRECT Corp., Cupertino, CA) filled with isoproterenol (Sigma, I5627) were implanted subcutaneously in AC6mut and control mice using 2% isoflurane/oxygen anesthesia. Infusion rate of isoproterenol was 60 mg/kg/d as previously described [7],[8] and was continued for seven days. Isoproterenol was dissolved in saline supplemented with 0.1% ascorbic acid as anti-oxidant. Pumps were removed a day prior to echocardiography and hemodynamic measurements to avoid the confounding effects of concurrent isoproterenol infusion on measurements of LV dimensions and function. All physiological studies were performed without knowledge of group identity.

### Echocardiography

Anesthesia was induced with 5% isoflurane (at a flow rate of 1 L/min oxygen) and maintained at 1% isoflurane in oxygen. Echocardiographic images were obtained using a 9-18L MHz linear probe and a Vevo 2100 Imaging system.

### *In vivo* physiology

Mice were anesthetized with sodium pentobarbital (80 mg/kg, ip) and a 1.4F conductance-micromanometer catheter (SPR 839, Millar Instruments, Houston, TX) was inserted *via* the right carotid artery across the aortic valve and into the LV chamber. After LV pressures were recorded, bilateral vagotomy was performed to minimize confounding effects of reflex activation. End-systolic pressure, and the rates of LV pressure development (LV +dP/dt) and decline (LV -dP/dt) were obtained. Inferior vena cava occlusion was performed to reduce LV volume for end-systolic pressure-volume relationship (ESPVR) measurements [9]. The product of stroke volume, determined by conductance catheter, and heart rate was used to determine cardiac output. Data were acquired and analyzed without knowledge of group identity.

### Cyclic AMP measurement

Isolated mice cardiac myocytes were stimulated with isoproterenol (10 μM, 10 min) or NKH477 a water-soluble forskolin analog (10 μM, 10 min), and then lysed (2.5% DTAB, 0.05 M sodium acetate, pH 5.8, and 0.02% bovine serum albumin). Supernatant cAMP was measured using the cAMP Biotrak Enzyme Immunoassay System (GE Healthcare) as previously reported. [1].

### PKA activity assay

Isolated cardiac myocytes were stimulated with isoproterenol (10 μM, 10 min) or NKH477 (10 μM, 10 min). Cardiac myocytes were homogenized in buffer A (20 mM Tris-HCl, pH 7.4–0.5 mM EGTA-0.5 mM EDTA, and protease inhibitor cocktail from Invitrogen) and centrifuged (14,000 x *g*, 5 min, 4°C). The supernatant was incubated with PKA biotinylated peptide substrate (SigmaTECH^®^ cAMP-Dependent Protein Kinase Assay System, Promega, Madison WI) in the presence of [γ-^32^P]-ATP. The ^32^P-labeled, biotinylated substrate was recovered with a streptavidin matrix, and the specific activity of PKA determined.

### Cardiac myocyte apoptosis

Cardiac myocyte apoptosis was determined using terminal deoxynucleotidyl transferase dUTP nick end labeling (TUNEL), as previously described [10]. Paraffin embedded heart tissue sections were deparaffinized and rehydrated using 100% Citrasolv followed by ethanol. Antigen retrieval and nuclear permeabilization were performed by submerging the slides in 10 mM citrate (pH 6.0) and heating by microwave. Nuclease-generated positive controls were obtained by treating tissue with TACS-nuclease in TACS-nuclease buffer (Trevigen, Gaithersburg, MD). TUNEL staining was performed using a CardioTACS kit except that the Streptavidin-HRP Solution was replaced with Streptavidin-Oregon Green 488 (Invitrogen, Carlsbad, CA). Finally, the slides were mounted using Prolong Gold with DAPI (Invitrogen, Carlsbad, CA), and imaged by fluorescence microscopy through a 40x lens (Delta Vision System). Blue nuclei and green apoptotic nuclei were counted using Metamorph software (Molecular Devices, Sunnyvale, CA) stipulating a nuclear area from 8 to 450 pixel. Twelve images containing 8,000–10,000 nuclei were counted for each LV sample. Apoptotic nuclei were expressed as percentage of total nuclei. Caspase 3/7 activity was determined as previously described [4].

### LV calcineurin activity

Calcineurin activity was assayed using the Calcineurin Cellular Activity Assay Kit (Enzo Life Sciences, Farmingdale NY). LV homogenates were placed in lysis buffer (provided by Enzo) and centrifuged at 16,000 x g (4°C, 60 min). Supernatant was desalted by gel filtration. Five micrograms of protein were used in the assay. The results are reported as the amount of phosphate released (nmol/microgram protein).

### Detection of mRNA and immunoblotting

Reverse transcription-quantitative polymerase chain reaction (RT-qPCR) was used to quantify mRNA and immunoblotting was used to quantify protein content. Primers for FHL1 (Forward: TGCAACAAGTGCGCTACTCG, Reverse: CAATGTTTGGCGAACTTGGTC); Periostin (Forward: GAGAGTACACCTTATTAGCACCTGTGTA, Reverse: GGTCGCTAAGGCCAACTTTTAC); and collagen 1 (Forward: GCCAAGAAGACATCCCTGAAG, Reverse: GGGTCCCTCGACTCCTAC).

Antibodies used in immunoblotting included: anti-FHL1 (Aviva Systems Biology, San Diego CA; 1:1,000), anti-Bcl2, anti-TnI and anti-phospho-TnI (Cell Signaling, Danvers MA; 1:1,000); anti-NFATC3 and anti-NFATC4 antibodies (Santa Cruz Biotechnology, Santa Cruz CA; 1:200), anti-PLB antibody (Affinity Bioreagents, Golden CO; 1:5,000); anti-phospho-S16-PLB antibody (Badrilla, Leeds UK; 1:3,000 dilution); anti-S100A1 (Novus Biologicals, Littleton CO; 1:1,000); anti-SERCA2a (Enzo Life Sciences, Farmingdale NY; 1:1,000); anti-GAPDH (Fitzgerald, Acton MA; 1:20,000); anti-periostin (R&D Systems, Minneapolis MN; 1:2,000); anti-phospho-CREB (Cell Signaling, Danvers MA; 1:1000). In all figures displaying immunoblotting, summary data are normalized to GAPDH.

### Statistical analysis

Data acquisition and analysis were done without knowledge of group identity. Group sizes were determined by power calculations. Data represent mean ± SE; 2-way ANOVA was used to detect differences among 4 groups. Sidak’s multiple comparison was used for between-group comparisons when ANOVA p<0.05. When two group comparisons were made, Student’s t-test was used (two-tailed). The null hypothesis was rejected when p<0.05. Analyses were performed using GraphPad Prism (GraphPad Software, Inc., San Diego CA).

## Results

### Echocardiography

Basal heart rates were similar in both groups before and showed similar decline after seven days of isoproterenol infusion (**Table 1**). LV end-diastolic dimension was unchanged by isoproterenol infusion in both groups. In contrast, end-systolic dimension increased similarly in both groups. LV ejection fraction was reduced by isoproterenol in both groups, but less so in mice with cardiac-directed AC6mut expression (p = 0.047).

**Table 1:** Echocardiography: effects of sustained isoproterenol infusion.

	*Control (10)*			*AC6 mutant (11)*			*ΔCon vs ΔAC6mut* *p*
	*Pre Iso*	*7d Iso*	*p*	*Pre Iso*	*7d Iso*	*p*	
*HR (bpm)*	506±9	466±14	<0.03	520±7	462±12	0.0005	0.50
*EDD (mm)*	3.8±0.1	3.7±0.1	0.49	3.9±0.2	3.9±0.1	1.0	0.87
*ESD (mm)*	2.2±0.1	2.9±0.1	0.0001	2.4±0.2	2.9±0.1	0.037	0.17
*LVEF (%)*	72±2	47±2	0.0001	70±3	53±3	0.0007	0.047

HR, heart rate; EDD, end-diastolic diameter; ESD, end-systolic diameter; LVEF, left ventricular ejection fraction. Values represent mean ± SE. *P* values are from Student’s *t*-test (2-tailed, paired) comparing changes for each variable within groups (columns 4 and 7) and between groups (final column; 2-tails, unpaired)

### LV contractile function

**Fig 1:**
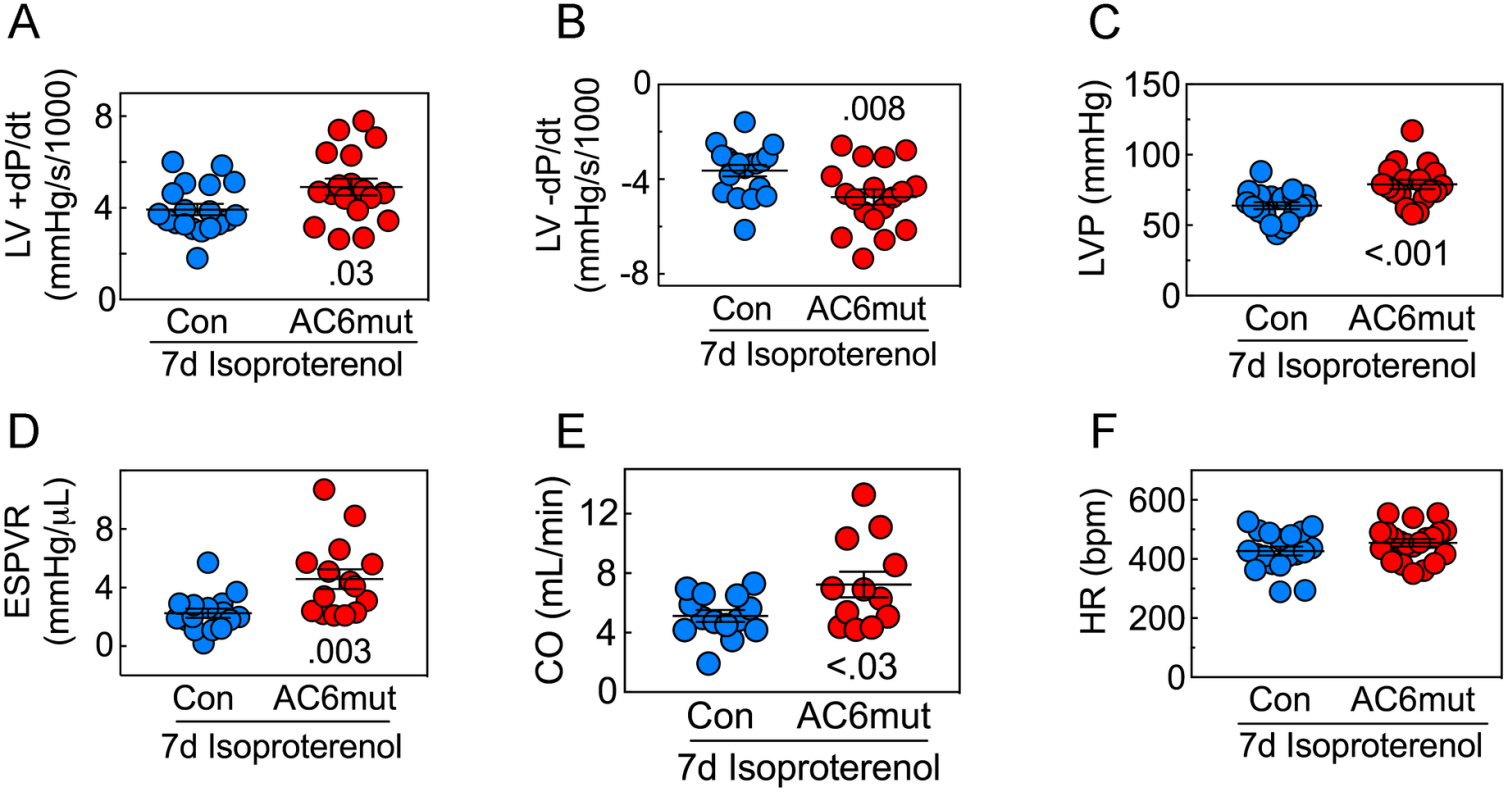
LV function. **A.** After seven days of continuous isoproterenol infusion, mice with cardiac-directed AC6mut expression exhibited a 25% greater LV peak +dP/dt (p = 0.03), indicating increased LV systolic function. **B.** LV peak -dP/dt was also superior in mice with cardiac-directed AC6mut expression (31% increase, p = 0.008), indicating increased LV diastolic function. **C.** LV developed pressure also was superior in mice with cardiac-directed AC6mut expression. **D.** The slope of the LV end-systolic pressure-volume relationship (ESPVR), a load-independent measure of LV contractility, was also 2-fold steeper in mice with cardiac-directed AC6mut expression (p = 0.003) **E.** Cardiac output was 41% higher in AC6mut mice (p<0.03). **F.** Heart rate showed no group difference. Data are from individual animals, collected without knowledge of group identity; mean±SE are also indicated; p value from Student’s t-test, unpaired, 2-tailed.

For a more load-independent evaluation of LV contractile function than ejection fraction, we measured LV pressure development and decay (**Fig 1; S15–S17 Figs**). AC6mut mice showed superior LV developed pressure (p<0.001), LV peak +dP/dt (p = 0.03) and LV peak -dP/dt (*p* = 0.008). The mean slope of the ESPVR, perhaps the best measure of LV contractile function, was 2-fold steeper (p = 0.003) in AC6mut compared to control mice. Finally, AC6mut mice also showed a 41% greater basal cardiac output (p<0.03) than controls. There was no group difference in heart rate.

### Cyclic AMP, PKA activity, and Ca2+ handling proteins

**Fig 2:**
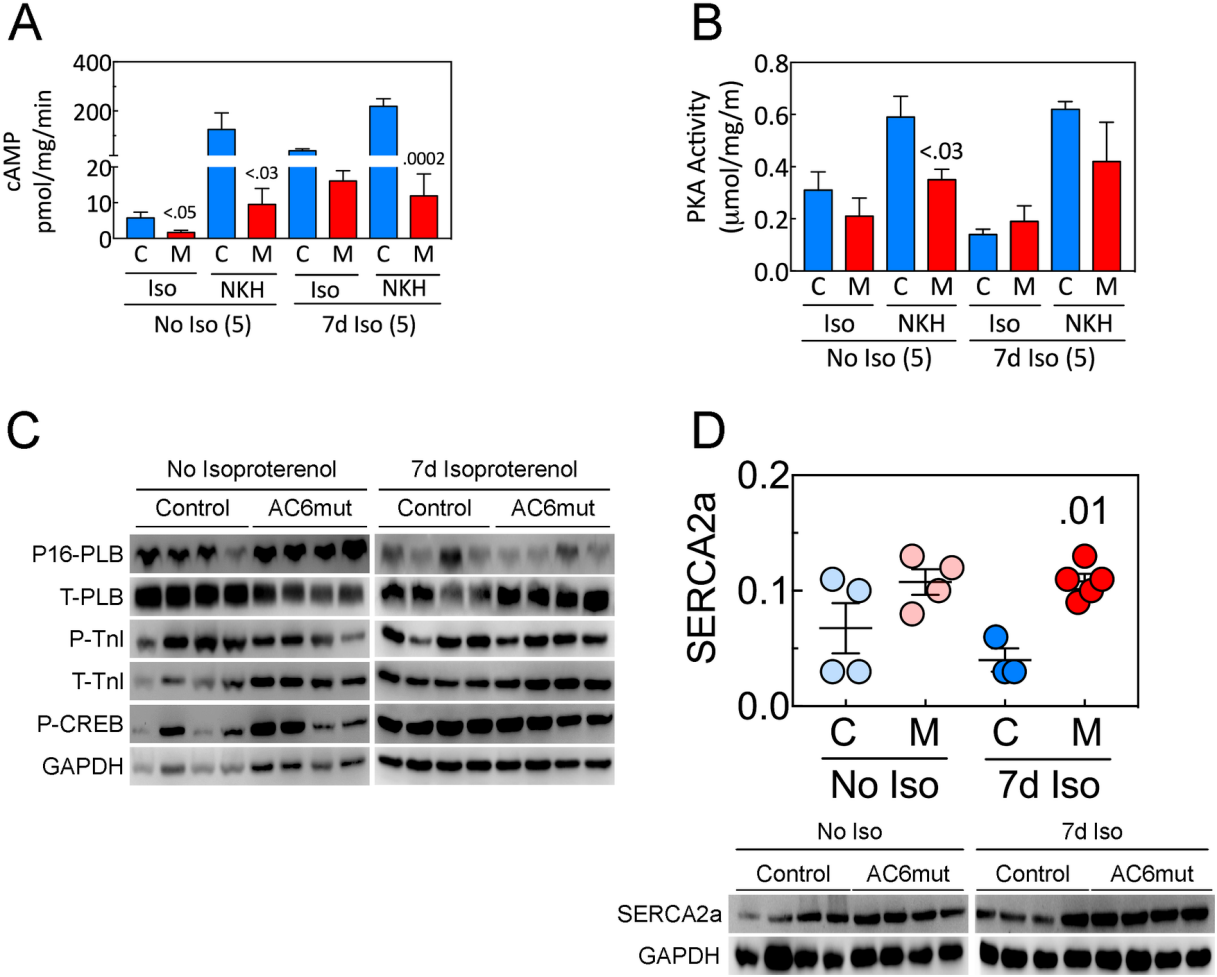
LV cAMP, PKA Activity and Signaling Proteins. **A.** *Cyclic AMP Generation*. LV membranes from AC6mut (M) and control mice (C) were used to measure cAMP production before (No Iso, n = 5) and after 7d isoproterenol infusion (7d Iso, n = 5). Membranes were stimulated with isoproterenol (Iso; 10 μM, 10 min) or NKH477 (NKH, a water-soluble direct AC stimulant; 10 μM, 10 min). Cyclic AMP production was lower in LV from AC6mut than from control mice before and after 7d isoproterenol infusion. **B.** *PKA Activity* in isolated cardiac myocytes before (No Iso, n = 5) and after 7d isoproterenol infusion (7d Iso, n = 5). Cardiac myocytes were stimulated with isoproterenol (Iso; 10 μM, 10 min) or NKH477 (NKH, 10 μM, 10 min). PKA activity (μmol/mg/min) was lower in cardiac myocytes from AC6mut (M) than from control mice (C) before isoproterenol infusion, but no group differences were seen after 7d isoproterenol infusion. **C.** There was no group difference in total or phosphorylated PLB, total or phosphorylated troponin-I or CREB phosphorylation after 7d of continuous isoproterenol infusion. However, there were pre-Iso group differences in total PLB (reduced in AC6mut, p<0.003), and in P-TnI (reduced in AC6mut, p = 0.005). In these instances, 7d isoproterenol was associated with reduced levels in the control animals, such that there no longer were group differences. **D.** LV SERCA2a protein showed group differences (2-Way ANOVA: Interaction: p = 0.29; Iso: p = 0.44; Gene: p = 0.002). Seven days Iso increased LV SERCA2a in AC6mut (M) mice vs control (p = 0.01). In **A and B**, P values from Student’s t-test (unpaired, 2-tailed); In **D**, p value from 2-way followed by Sidak’s multiple comparison test. In all Figures displaying immunoblotting, summary data are normalized to GAPDH.

Increased AC6mut expression was associated with reduced cAMP generation in normal cardiac myocytes as we reported in our previous study [5]. Before and after seven days of isoproterenol infusion, we determined cAMP levels in cardiac myocytes isolated from AC6mut or control mice (**Fig 2A; S7 Fig**). Isoproterenol-stimulated cAMP production was lower in LV samples from AC6mut than from control mice before isoproterenol infusion (p<0.05). In addition, direct AC stimulation of cAMP was lower in cardiac myocytes from AC6mut mice both before (p<0.03) and after 7d isoproterenol infusion (95% reduction, p = 0.0002).

PKA activity in cardiac myocytes isolated from AC6mut or control mice was also determined before and after seven days of isoproterenol infusion (**Fig 2B; S8 Fig**). AC-stimulated PKA activity (NKH) was lower in cardiac myocytes from AC6mut than from control mice before isoproterenol infusion (p<0.03; **Fig 2B; S8 Fig**), but no group differences were seen after 7d isoproterenol infusion.

Cardiac-directed AC6mut expression had no effects on PKA substrates such as phosphorylation of phospholamban (PLB) at Ser16, troponin I (TnI) at Ser22/23, or cAMP-responses element binding protein (CREB) at Ser133 after 7d isoproterenol infusion (**Fig 2C**).

However, there were pre-Iso group differences in total PLB (reduced in AC6mut, p<0.003), and in P-TnI (reduced in AC6mut, p = 0.005). In these instances, 7d isoproterenol was associated with reduced levels in the control animals, such that there no longer were group differences. **Table 2**shows quantitative analysis of immunoblotting data in **Fig 2C**.

**Table 2:** LV protein expression quantitative analysis (Fig 2C).

	*No Iso*		*7d Iso*		*p*		
	*Control (3–4)*	*AC6mut (4)*	*No Iso (4)*	*7d Iso (4)*	Gene	Iso	Inter
*p-PLB*	1.4±.3^A^	1.8±.1^B^	.3±.1	.2±.1	ns	< .0001	ns
*Total PLB*	2.7±.7^C^	.7±.1	.5±.08^D^	.81±.11	.01	.004	< .002
*p-TnI*	.86±.17	.24±.05^E^	.37±.1^F^	.4±.1	< .01	ns	.01
*Total TnI*	.1±.02^G^	.1±.01	.1±.01	.1±.01	ns	.0005	ns
*p-CREB*	.4±.2	.6±.2	.8±.03	.7±.01	ns	.034	ns
*FHL1*	1.6±.1	.6±.1	4.7±.7	1.6±.3	.0001	.0001	< .02

^A^p<0.002 vs Control 7d Iso

^B^p = .00001 vs AC6mut 7d Iso

^C^p<0.003 vs AC6mut No Iso

^D^p = 0.004 vs Control No Iso

^E^p = 0.005 vs Control No Iso

^F^p = 0.025 vs Control No Iso

^G^p = 0.004 vs Control 7d Iso.

Values (arbitrary densitometry units) from 4 independent groups of mice. Data are mean ± SE; P values from 2-way ANOVA. Subsequent within- and between-group comparisons (when ANOVA p<0.05) were conducted using via Sidak’s multiple comparison test. Iso, isoproterenol; 7d Iso, 7d continuous isoproterenol infusion; PLB, phospholamban; p-PLB, phosphorylated PLB; TnI, troponin I; p-TnI, phosphorylated TnI, p-CREB, phosphorylated cAMP response element binding protein

In contrast, SERCA2a protein expression was higher in LV from AC6mut mice than in control mice after 7d sustained isoproterenol infusion (p<0.01, **Fig 2D; S9 Fig**).

### Calcineurin activity, NFAT phosphorylation & FHL1 expression

**Fig 3:**
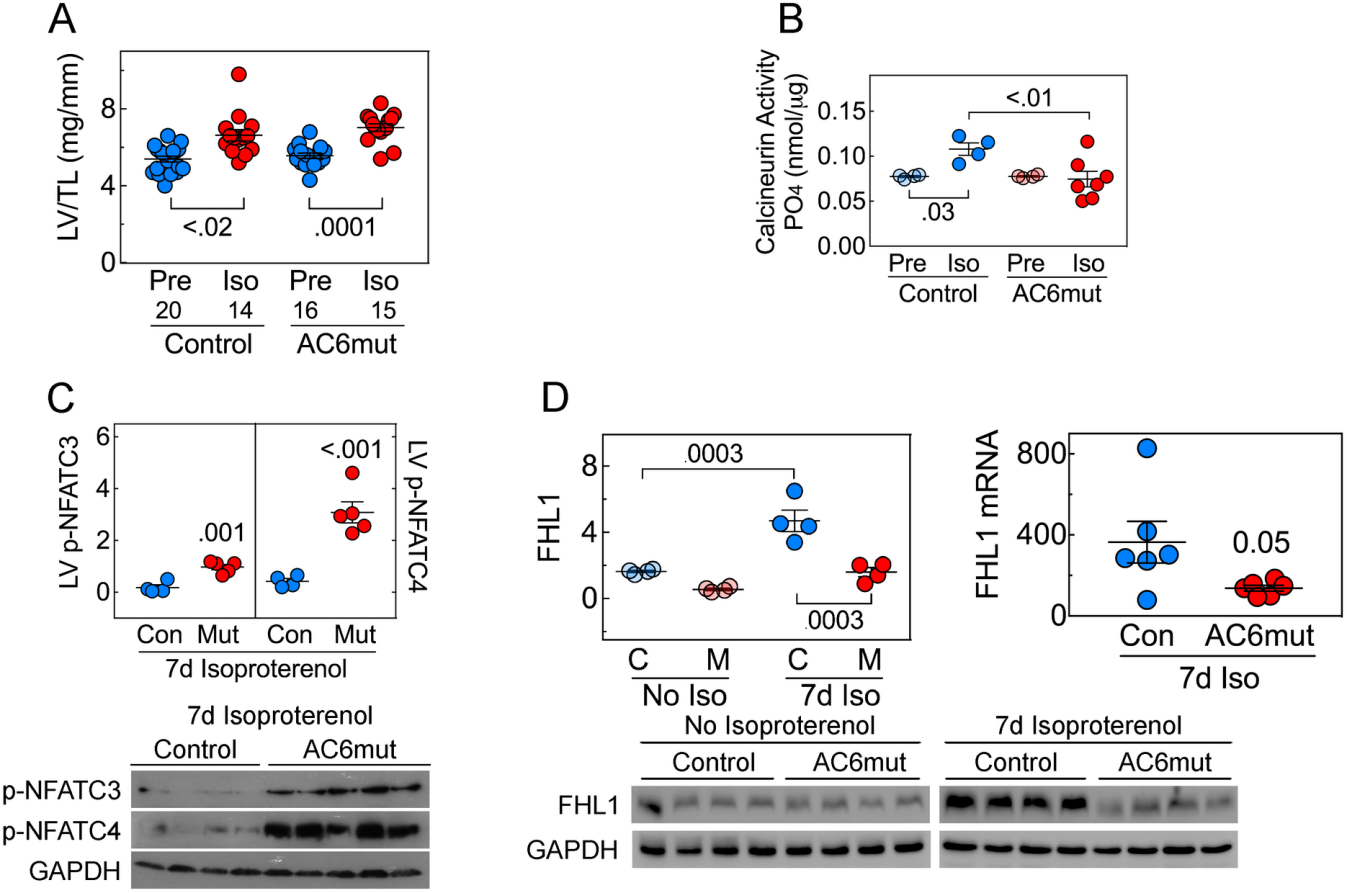
LV hypertrophy. **A.** LV-to-Tibial length (LV/TL) ratios were increased by 7d isoproterenol in both groups (2-Way ANOVA: Interaction: p = 0.80; Iso: p<0.0001; Gene: p = 0.43) and differences were seen within groups (Control: p<0.02; AC6mut: p = 0.0001; Sidak’s multiple comparison test). There were no between-group differences before or 7d after continuous isoproterenol infusion. **B.** Calcineurin activity (PO4 release) in LV samples showed an overall group difference (2-way ANOVA: Interaction: p<0.04; Iso: p = 0.085; Gene: p = 0.04). A 7d isoproterenol infusion resulted in increased calcineurin activity in control mice (p = 0.03), not seen in AC6mut mice; AC6mut mice showed lower calcineurin activity vs control mice after 7d isoproterenol (p<0.01). Between-group comparisons were made using Sidak’s multiple comparison test. **C.** LV phosphorylation of NFATC3 (p = 0.001) and NFATC4 (p<0.001) were higher after isoproterenol infusion in AC6mut (Mut) mice. **D.** LV FHL1 protein content (L graph) was 3-fold greater in control than in AC6mut mice 7d after isoproterenol infusion (p<0.01), although FHL1 content was similar in both groups before isoproterenol infusion. LV FHL1 mRNA (R graph) was higher in control than in AC6mut mice after 7d isoproterenol. In C & D, p values are from Student’s t-test (unpaired, 2-tailed). In Figures displaying immunoblotting, summary data are normalized to GAPDH.

LV-to-tibial length (LV/TL) ratios were increased by 7d isoproterenol (2-Way ANOVA: Interaction: p = 0.80; Iso: p<0.0001; Gene: p = 0.43), and differences were seen within groups (control: p = 0.02; AC6mut: p = 0.0001) (**Fig 3A; S10 Fig**). There were no between-group differences before or 7d after continuous isoproterenol infusion. LV calcineurin activity showed no group difference prior to isoproterenol infusion. However, 7d of continuous isoproterenol infusion was associated with a 39% increase in LV calcineurin activity in control mice (p = 0.03) while AC6mut mice showed no change (**Fig 3B; S11 Fig**). AC6mut mice showed lower calcineurin activity vs control mice after 7d isoproterenol (p<0.01). In addition, after 7d isoproterenol infusion, Nuclear Factor of Activated T-Cells 3 (NFATC3) (p = 0.001) and NFATC4 phosphorylation (p<0.001) were greater in LV samples from AC6mut mice vs control (**Fig 3C; S12 Fig**). Sustained isoproterenol infusion was associated with lower levels of Four and a Half LIM domains protein 1 (FHL1) protein in LV samples from AC6mut mice (p<0.01), although no group difference was present prior to isoproterenol (**Fig 3D; S13 Fig**). LV *FHL1* mRNA expression also was reduced in AC6mut mice after isoproterenol infusion (**Fig 3D; S14 Fig**).

### Cardiac myocyte apoptosis

**Fig 4:**
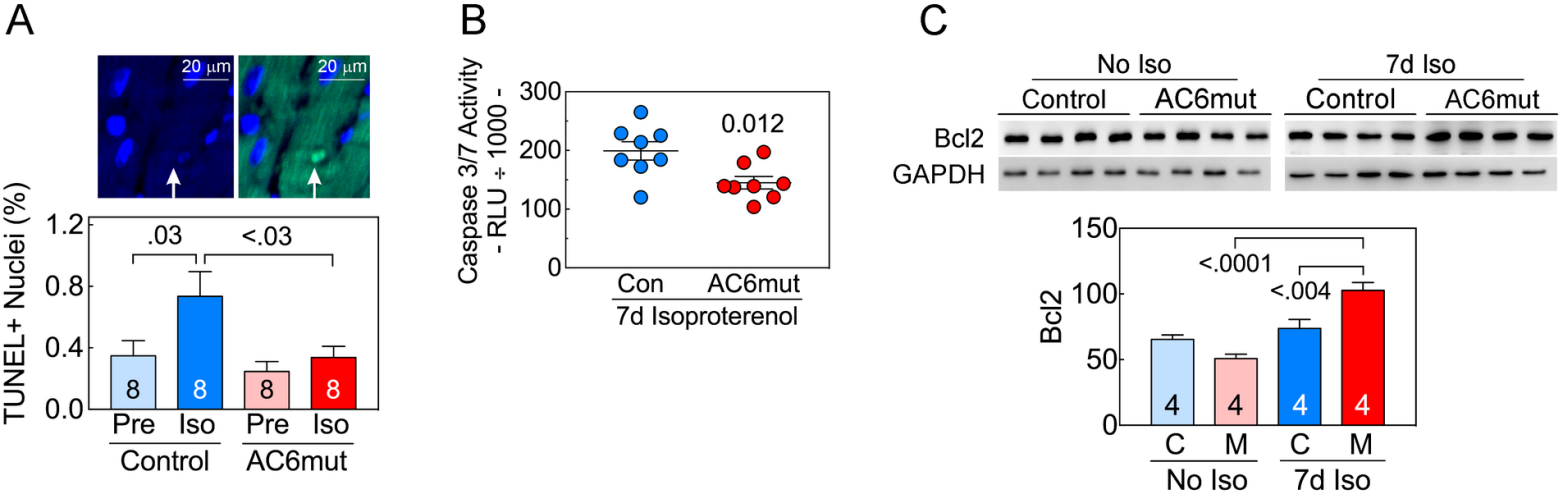
Cardiac myocyte apoptosis. **A.** *Upper Panel*: Example of TUNEL positive nucleus in photomicrographs from confocal microscopic images of transmural LV sections. Nuclei were stained with DAPI (blue) and apoptotic nuclei with broken DNA were labeled with FITC (green). *Lower Panel*: Quantitative analysis of cardiac myocyte apoptosis in AC6mut vs control mice before (Pre) and after continuous isoproterenol infusion showed group differences (2-Way ANOVA: Interaction: p = 0.17; Iso: p = 0.03; Gene: p<0.03). Cardiac myocyte apoptosis was increased >2.1-fold in control mice after isoproterenol infusion (p = 0.03), but was not increased in LV samples from mice with cardiac-directed AC6mut expression. Control mice showed more apoptosis vs AC6mut mice after 7d isoproterenol (p<0.03). Between-group comparisons made using Sidak’s multiple comparison test. **B.** After 7d isoproterenol infusion, LV caspase 3/7 activity was greater in control mice (Con) than in AC6mut mice (p = 0.012, Student’s t-test, unpaired, 2-tailed). **C.** LV Bcl2 protein content showed group differences (2-Way ANOVA: Interaction: p = 0.001; Iso: p<0.0001; Gene: p = 0.19). Seven days after continuous isoproterenol infusion, LV Bcl2 was increased in AC6mut mice (M), compared to control mice (p<0.0001). Bcl2 was increased after 7d isoproterenol infusion in AC6mut (p<0.004). Between-group comparisons made using Sidak’s multiple comparison tests. In all graphs mean ±SE are shown. In C, summary data are normalized to GAPDH.

There was a group difference in TUNEL-positive nuclei after 7d isoproterenol infusion. Control mice showed a 2.1-fold increase in LV apoptotic nuclei (p = 0.03; **Fig 4A; S15 Fig**), while AC6mut mice showed no change. Control mice show more apoptosis vs AC6mut mice after 7d isoproterenol (p<0.03). LV caspase 3/7 activity was 1.3-fold higher in control mice after isoproterenol infusion (p = 0.012, **Fig 4B; S16 Fig**). AC6mut expression was associated with increased Bcl2 protein (p<0.0001; **Fig 4C; S17 Fig**). Bcl2 was also increased after 7d isoproterenol in AC6mut mice (p<0.004). LV Bax protein expression showed no group difference (data not shown).

### LV fibrosis

**Fig 5:**
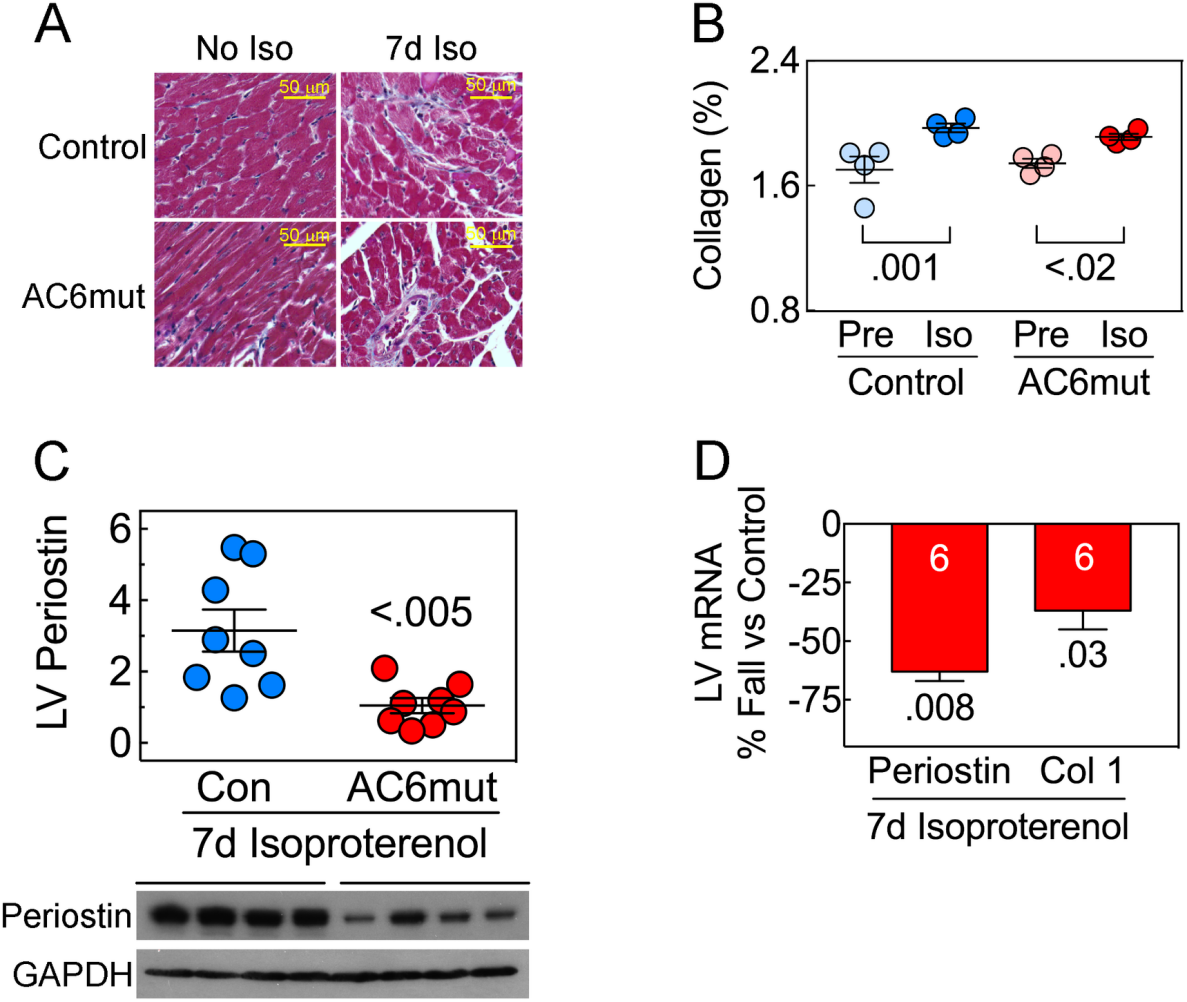
LV fibrosis. **A.** LV collagen deposition LV sections using Masson trichrome staining. **B.** LV collagen deposition showed group differences (2-Way ANOVA: Interaction: p = 0.30; Iso: p<0.0001; Gene: p = 0.30). Collagen deposition was increased following 7d isoproterenol in control (p = 0.001) and in AC6mut mice (p<0.02). Between-group comparisons made using Sidak’s multiple comparison test. **C.** LV periostin protein expression was greater in control mice than in AC6mut mice after 7d isoproterenol infusion (p<0.005). Periostin protein was undetectable prior to isoproterenol infusion. **D.** LV mRNA expression of periostin, and collagen 1 (Col 1) were detected using RT-PCR. Compared to control mice, 7d isoproterenol infusion was associated with reduced LV periostin mRNA (p = 0.008) and reduced collagen-1 mRNA (p = 0.03; data normalized to GADPH mRNA). In C & D, p values from Student’s t-test (unpaired, 2-tailed).

In both groups, 7d isoproterenol infusion was associated with a small but significant increases in LV fibrosis (control: p = 0.001; AC6mut: p<0.02; **Fig 5A and 5B; S18 Fig**). Periostin protein was 3-fold lower in LV from AC6mut mice vs control mice after 7d isoproterenol infusion (p<0.005; **Fig 5C; S19 Fig**), which correlated with a similar decrease in LV *periostin* mRNA (p = 0.008; **Fig 5D; S20 Fig**). Finally, after 7d isoproterenol LV from AC6mut mice showed lower *collagen-*1 mRNA expression (p = 0.03; **Fig 5D; S20 Fig**). Periostin expression was undetectable by immunoblotting prior to sustained isoproterenol infusion. However, mRNA levels of periostin prior to sustained isoproterenol infusion showed no group difference (Control: 1.0± 0.14; AC6mut: 0.84±0.07; n = 4, both groups; p = 0.34).

### Necropsy

**Table 3**shows data obtained at necropsy from four independent groups of mice. Isoproterenol infusion for seven days was associated with an increase in LV weight and LV weight-to-tibial length ratio in control and AC6mut mice (**Fig 3A; S10 Fig** and **Table 3**). Liver weight, normalized to tibial length showed no group differences. Lung weight-to-tibial length was increased in AC6mut mice that received 7d isoproterenol. However, left atrial weight-to-tibial length ratios were increased after 7d isoproterenol in control mice (p<0.001) but not in AC6mut mice. Finally, there were similar non-significant increases in right ventricular weight-to-tibial length ratios after 7d isoproterenol in both groups.

**Table 3:** Necropsy.

	*Control*		*AC6mut*		*p*		
	*No Iso (20)*	*7d Iso (14)*	*No Iso (16)*	*7d Iso (15)*	Gene	Iso	Inter
*BW (g)*	25.9±1.1	24.1±0.7	24.6±0.6	26.1±0.7	0.69	0.86	0.06
*Tibial length (mm)*	16.8±0.2	16.6±0.2	17.0±0.1	17.5±0.3	0.01	0.48	0.10
*LV (mg)*	90.8±3.1	110.2±5.3^A^	94.9±3.1	123.1±4.3^B^	0.03	<0.0001	0.26
*LV/TL (mg/mm)*	5.4±0.2	6.6±0.3^C^	5.6±0.1	6.7±0.1^D^	0.43	<0.0001	0.80
*Liver/TL (mg/mm)*	68±3	76±3	74±3	77±4	0.29	0.10	0.45
*Lung/TL (mg/mm)*	8.9±0.3	9.1±0.2	8.9±0.2	10.0±0.2^E^	0.07	0.01	0.07
*LA/TL (mg/mm)*	0.21±0.02	0.33±0.03^F^	0.22±0.01	0.27±0.02	0.24	0.0001	0.10
*RV/TL (mg/mm)*	1.1±0.1	1.4±0.1	1.2±0.1	1.5±0.1	0.33	<0.005	0.99

^A^p = 0.0002 vs Control No Iso

^B^p<0.0001 vs AC6mut No Iso

^C^p<0.02 vs Control No Iso^;^

^D^p = 0.001 vs AC6mut No Iso

^E^p<0.02 vs AC6mut No Iso

^F^p<0.001 vs Control No Iso.

Values from 4 independent groups of mice. Data are mean ± SE; P values from 2-way ANOVA. Subsequent within- and between-group comparisons (when ANOVA p<0.05) were conducted using via Sidak’s multiple comparison test. Iso, 7d continuous isoproterenol infusion; BW, body weight; LV, left ventricle; TL, tibial length; LA, left atrial; RV, right ventricular; Iso, isoproterenol

## Discussion

The most important conclusion from our studies is that mice with cardiac-directed expression of AC6mut weather the deleterious effects of continuous isoproterenol infusion considerably better than control mice. First, after seven days of isoproterenol infusion, mice with cardiac-directed AC6mut expression exhibited superiority (vs control mice) in peak +dP/dt, peak -dP/dt, the slope of the ESPVR, and cardiac output, which are measures of systolic, diastolic and contractile function. Second, AC6mut mice, unlike control mice, did not show the expected increase in cardiac myocyte apoptosis following isoproterenol infusion. These findings indicate that AC6mut expression protects the heart against β-adrenergic receptor stimulation, and does so while preserving LV systolic and diastolic function despite lower levels of cardiac cAMP. These findings provide a rationale for proceeding to additional preclinical studies—to see whether AC6mut gene transfer has beneficial effects in pre-existing HF.

### AC6mut transgenic line

The AC6mut transgenic line that we used produces a 17-fold increase in cardiac AC6mut protein (vs endogenous AC6) distributed to plasma membrane and cytosolic compartments [5], thereby reducing cell-surface transduction of β-adrenergic receptor-mediated signaling, as well influencing protein:protein interactions within the cell. Although endogenous AC6 expression is unaltered in the AC6mut line [5], excess AC6mut likely played a dominant negative role in competing for Gsα and thereby impeding endogenous AC6 activation, thus explaining the 3.5-fold reduction in isoproterenol-stimulated cAMP production in LV from AC6mut mice (**Fig 2A; S7 Fig**).

In a previous publication describing the effects of targeted deletion of AC6, which reduced LV cAMP generating capacity to a similar degree as does AC6mut expression, we found that limiting cAMP production did not prevent isoproterenol-induced cardiomyopathy, indicating that the pathogenesis of cardiomyopathy does not require sustained increases in cardiac cAMP levels. In the present study, the relative preservation of LV peak +dP/dt and peak -dP/dt in AC6mut mice vs control after sustained isoproterenol infusion (**Fig 1A, S1 Fig; Fig 1B, S2 Fig**), is likely due to the presence of cAMP-independent beneficial effects.

### Group differences in LV function

The 7d isoproterenol infusion that we used in the current study had deleterious effects on LV function in control mice that are similar to what we and others previously have reported [7],[8],[11][12]. For example, a 4d isoproterenol infusion of 60 mg/kg/d (42 μg/kg/min) resulted in similar decrements in ejection fraction and increases in end-systolic diameter in normal C57B6 mice [12] to those we report here after a 7d isoproterenol infusion. Previous studies of isoproterenol infusion of 7–14 days show consistent time-related increases in LV mass, which vary somewhat by concentration and duration of infused isoproterenol, and whether data are reported as total heart weight or LV weight normalized to body weight or to tibial length [7],[8],[11] [12].

Seven days of isoproterenol infusion caused dilation of LV end-systolic diameter that was similar in both groups (**Table 1**). However, the fall seen in LV ejection fraction after 7d isoproterenol infusion was less among AC6mut mice (Con: 26±2 percentage units; AC6mut: 17±3 percentage units; p = 0.047) a 31% relative reduction. This protective effect of AC6mut expression was mirrored in the AC6mut effect on LV +dP/dt (25% higher than control, p = 0.03, **Fig 1A; S1 Fig**) and LV -dP/dt (38% higher than control, p = 0.008, **Fig 1B; S2 Fig**). The ESPVR, perhaps the best measure of contractile function because of its relative independence of LV loading conditions [13], showed a pronounced group difference—mean values for AC6mut mice were 2-fold steeper than those in control mice (p = 0.003, **Fig 1D; S4 Fig**). Changes of this magnitude would be anticipated to have an important physiological effect in clinical heart failure. The slope of the ESPVR in human subjects with dilated cardiomyopathy was reported to be reduced by 35% vs subjects with normal LV function [14], so a doubling of slope of the ESPVR, as we report here, if translated to clinical settings, would restore normal function. Similar decrements of LV peak +dP/dt have been reported in patients with heart failure in previous studies [15],[16]

We previously reported that cardiac-directed AC6mut expression was associated with normal LV contractile response to acute isoproterenol stimulation despite impairment of cAMP generating capacity through changes in LV Ca2+ handling [5]. In that previous study, which did not employ sustained isoproterenol infusion, AC6mut expression was associated with increased peak Ca2+ transients, and favorable alterations in LV Ca2+ handling proteins [5]. In the present study, although we did not assess Ca2+ transients, SERCA2a, a key Ca2+ handling protein, was expressed at a 2.7-fold higher level in LV from AC6mut than control mice 7d after sustained isoproterenol infusion (**Fig 2D; S9 Fig**). The beneficial effects conferred by AC6mut attenuated the deleterious effects of isoproterenol, leading to group differences in LV function and decreased apoptosis, each with correlative molecular signaling events that provide mechanistic insight.

Cardiac function in the AC6mut line appears to contradict the usual close relationship between cAMP generating capacity and LV function. For example, a 50% reduction in LV cAMP is seen in severe HF in multiple preclinical models, with a proportional decrement in LV function [17,18]. Furthermore, AC6 deletion reduces LV cAMP generating capacity and LV function proportionately [4]. A striking finding in the present study is that despite a marked reduction in cAMP generating capacity associated with AC6mut expression, LV function, compared to control mice, is unimpaired before [5], and superior to control mice after sustained isoproterenol infusion (**Fig 1A, 1B and 1D; S1, S2 and S4 Figs**).

### Isoproterenol-related LV hypertrophy

Increases in LV mass occur with even brief administration of isoproterenol. In the present study after a 7d isoproterenol infusion, we saw a 22% increase in LV-to-tibial length ratio in control mice, and a 20% increase in AC6mut mice (**Fig 3A, S10 Fig;** and **Table 3**). The infusion caused an increase in calcineurin activity in control mice, which cardiac-directed AC6mut expression mitigated (**Fig 3B; S11 Fig**). Calcineurin decreases phosphorylation of NFATC3, one of the transcription factors in the NFAT family, and thereby promotes NFATC3 entry into the nucleus, which then activates the hypertrophic gene program [19],[20]. The attenuation of isoproterenol-related increases in LV calcineurin activity among AC6mut mice was associated with increased NFATC3 phosphorylation (**Fig 3B and 3C: S11 and S12 Figs**), which would be predicted to reduce its nuclear entry. The importance of NFATC4 translocation and its role in the attenuation of hypertrophy is less well established. We also saw reduced LV FHL1 after isoproterenol infusion in AC6mut mice (**Fig 3D; S13 Fig**). FHL1 plays an important role in stress-induced LV hypertrophy and its deletion attenuates pressure-induced LV hypertrophy [21].

Despite favorable effects that would be predicted to attenuate hypertrophy (reduced calcineurin activity, increased NFAT, reduced FHL1 expression), there were no group differences in LV hypertrophy after sustained isoproterenol infusion. However, if these potentially beneficial alterations are present when AC6mut mice are challenged by other models of HF (myocardial infarction-induced HF, pressure stress), reduction in hypertrophy may be seen. Additional studies are needed to address this possibility.

We saw an increase in lung weight-to-tibial length ratio in AC6mut mice (**Table 3**) 7d after isoproterenol infusion, a finding that can indicate lung congestion. However, left atrial weight-to tibial length ratio, an indicator of left atrial enlargement, was increased only in control mice after 7d isoproterenol, making the difference in lung weight less likely a consequence of congestion.

### Cardiac myocyte apoptosis

Sustained infusion of isoproterenol induces cardiac myocyte apoptosis. We found a 2-fold increase in apoptotic rate after 7d isoproterenol infusion in control animals (**Fig 4A; S15 Fig**), an increase that is similar to what others have reported using similar doses and duration of isoproterenol infusion [22],[23]. There was a corresponding increase in caspase 3/7 activity (**Fig 4B; S16 Fig**) and reduced Bcl2 expression (**Fig 4C; S17 Fig**). All of these changes were abrogated by cardiac-directed AC6mut expression (**Fig 4A, 4B and 4C: S15–S17 Figs**). Since cardiac myocyte apoptosis is deleterious in the failing heart [24], and sustained β-adrenergic-receptor stimulation is common in patients with heart failure, reducing β-adrenergic-receptor-stimulated apoptosis would be predicted to benefit the failing heart.

### Cardiac fibrosis

We saw reduced expression of periostin protein (**Fig 5C; S19 Fig**) and mRNA (**Fig 5D; S20 Fig**), and reduced *collagen 1* mRNA (**Fig 5D; S20 Fig**) in AC6mut mice compared to control mice following sustained isoproterenol infusion—changes that would be predicted to reduce fibrosis. However, there were similar modest increases in fibrosis in both groups after isoproterenol infusion (**Fig 5A and 5B; S18 Fig**). It is plausible that isoproterenol infusion for a longer period or, alternatively, pressure stress, may result in group differences in fibrosis, given these changes in LV *periostin* and *collagen 1* expression. Periostin is an extracellular matrix protein that is expressed and secreted by cardiac fibroblasts. Pathological stress, such as pressure overload or myocardial infarction, increases cardiac periostin expression. Periostin, by regulating collagen deposition, alters the mechanical properties of tissues. Cardiac-directed expression of periostin increases LV collagen deposition and aging-associated LV hypertrophy [25]. In contrast, periostin deletion decreases cardiac hypertrophy and fibrosis after pressure overload [25]. We previously reported increased periostin expression in AC6 deleted mice after transaortic constriction, which provides a pressure stress on the heart [11]. The precise mechanism for how AC6mut reduces LV periostin expression—and its implication in other models of HF—will require additional studies.

### C1C2 vs AC6mut

In the present study, mice with cardiac-directed AC6mut expression were less susceptible to the toxic effects of isoproterenol, with relative preservation of both systolic and diastolic function. The putative mechanism for improved LV function was likely, at least in part, a result of a 2.7-fold increase in LV SERCA2a expression, an effect that may have increased LV function despite reduced cAMP production. Similar findings were described in cardiac-directed expression of the cytoplasmic domains (C1C2) of AC6, a smaller molecule that, absent the two transmembrane moieties of the intact AC6 molecule, is isolated from the plasma membrane, and present inside the cell [26]. However, unlike AC6mut, C1C2 retains catalytic activity. C1C2 expression, similar to AC6mut, did not increase phosphorylation of phospholamban or troponin I, but increased SERCA2a by 2.6-fold and protected the heart from isoproterenol toxicity. However, cAMP-generating capacity is greater in C1C2 mice than in AC6mut mice, and the two have not been compared to determine their relative in efficacy in treating HF using gene transfer.

### AC6mut gene transfer

The logical next step toward the translation of the present studies to clinical application is additional preclinical studies testing whether AC6mut gene transfer is beneficial in pre-existing HF. In a previous study, we examined the effects of AC6mut expression in cultured cardiac myocytes using adenovirus-mediated gene transfer [3]. AC6mut gene transfer resulted in marked reduction in cAMP generating capacity and reductions in apoptosis and phenylephrine-induced cardiac myocyte hypertrophy. The effects of AC6mut vs AC6 gene transfer were similar, indicating that beneficial effects do not require increased cAMP generation [3]. Further studies will determine whether the beneficial effects seen after AC6mut gene transfer in cultured cardiac myocytes also occur after gene transfer in the intact failing heart.

### Limitations of present study

A potential limitation of the present study is that we did not examine Ca2+ transients. Our focus instead was on the deleterious effects of sustained isoproterenol infusion on function and structure of the intact heart. However, a 2.7-fold higher level of LV SERCA2a in AC6mut mice after Iso (**Fig 2D; S9 Fig**) would be predicted to have important effects on Ca2+ transients [27], as we have previously reported in AC6mut mice [5]. Finally, although the AC6mut line prevented some of the deleterious effects of isoproterenol infusion, translation to clinical studies will require treating pre-existing HF using AC6mut gene transfer.

### Conclusion

Cardiac-directed expression of AC6mut enabled mice to weather the deleterious effects of continuous isoproterenol infusion considerably better than control mice. AC6mut expression had beneficial effects on LV systolic, diastolic, and contractile function, and it reduced apoptosis. These findings indicate that AC6mut expression protects the heart against some aspects of β-adrenergic receptor stimulation, and does so while preserving LV systolic and diastolic function despite lower levels of cardiac cAMP. These findings provide a rationale for proceeding to the next logical preclinical study—to see whether AC6mut gene transfer has benefits in pre-existing heart failure.

## Supporting information

S1 Fig Fig 1A. (PZFX)

S2 Fig Fig 1B. (PZF)

S3 Fig Fig 1C. (PZFX)

S4 Fig Fig 1D. (PZFX)

S5 Fig Fig 1E. (PZFX)

S6 Fig Fig 1F. (PZFX)

S7 Fig Fig 2A. (PZFX)

S8 Fig Fig 2B. (PZFX)

S9 Fig Fig 2D. (PZFX)

S10 Fig Fig 3A. (PZFX)

S11 Fig Fig 3B. (PZF)

S12 Fig Fig 3C. (PZFX)

S13 Fig Fig 3D (L). (PZFX)

S14 Fig Fig 3D (R). (PZFX)

S15 Fig Fig 4A. (PZFX)

S16 Fig Fig 4B. (PZFX)

S17 Fig Fig 4C. (PZFX)

S18 Fig Fig 5B. (PZF)

S19 Fig Fig 5C. (PZFX)

S20 Fig Fig 5D. (PZFX)
